# Milk and Dairy Products: Good or Bad for Human Bone? Practical Dietary Recommendations for the Prevention and Management of Osteoporosis

**DOI:** 10.3390/nu13041329

**Published:** 2021-04-17

**Authors:** Alicja Ewa Ratajczak, Agnieszka Zawada, Anna Maria Rychter, Agnieszka Dobrowolska, Iwona Krela-Kaźmierczak

**Affiliations:** Department of Gastroenterology, Dietetics and Internal Diseases, Poznan University of Medical Sciences, 61-701 Poznan, Poland; aga.zawada@gmail.com (A.Z.); a.m.rychter@gmail.com (A.M.R.); agdob@ump.edu.pl (A.D.)

**Keywords:** cow’s milk, plant milk, osteoporosis, bone mineral density, lactose intolerance, cow’s milk allergy, nutrition, osteoporosis, bone health

## Abstract

Osteoporosis affects women twice as often as men. Additionally, it is estimated that 0.3 million and 1.7 million people have hip fractures in the USA and Europe, respectively. Having a proper peak bone mass and keeping it as long as possible is especially important for osteoporosis prevention. One of the most important calcium sources is milk and dairy products. Breast milk is the best infant food, but milk should not be avoided later in life to prevent losing bone mass. On the other hand, more and more people limit their milk consumption and consume other dairy or non-dairy products. For example, they are usually replaced with plant beverages, which should be consumed carefully in several age groups. Additionally, an important element of milk and dairy products, as well as plant beverages, are probiotics and prebiotics, which may modulate bone turnover. Dietary recommendations focused on milk, and dairy products are an important element for the prevention of osteoporosis.

## 1. Introduction

**Figure 1:**
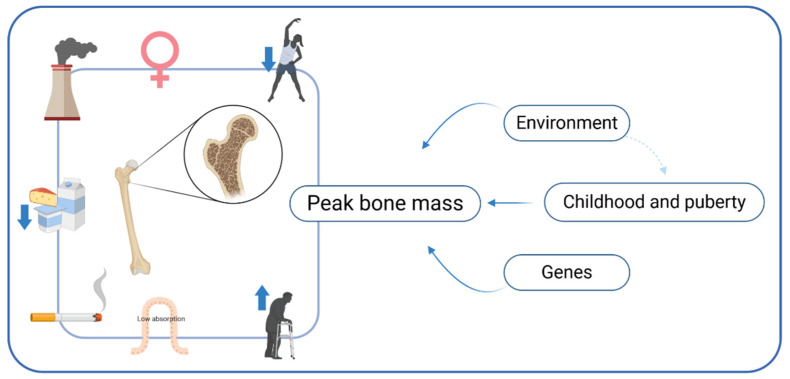
Risk factors of osteoporosis.

Osteoporosis is a skeletal disorder with decreased bone mineral density (BMD) and bone strength, leading to increased risk of fractures. Osteoporosis may be divided into primary and secondary (70% and 30% of all cases, respectively). Secondary osteoporosis can be caused by several diseases, e.g., inflammatory bowel diseases, celiac disease, or endocrinology disorders [1]. Risk factors of osteoporosis are, among others, malabsorption, cigarette smoking, stress, air pollution, older age, low physical activity, and co-occurring diseases (Figure 1) [2,3]. Osteoporosis affects women twice as often as men. Additionally, it is estimated that 0.3 million and 1.7 million people have hip fractures in the USA and Europe, respectively [4]. Having a proper peak bone mass and maintaining it as long as possible is especially important for osteoporosis prevention.

It is vital to note that 1/3 adult people achieve their total bone mass between 2 and 4 Tanner stages, and 95% of peak bone mass is reached before the age of 16. For this reason, puberty is a key time for bone mass formation [5,6]. Data about the age of peak bone mass are inconsistent, and it is suggested that a peak bone mass is reached at around 18 years of age for women and 20 years of age for men [7]. However, other authors suggest peak bone mass is reached between 20 and 30 years of age [8].

Additionally, peak bone mass is influenced by genetic and environmental factors, including diet [9]. Therefore, proper intake of minerals and vitamins, especially vitamin D and calcium, is essential, especially in a period of rapid growth, such as childhood and adolescence. The next stage is bone remodeling, which leads to total rebuilding of the skeleton—once every ten years with no change in bone net weight. Proper intake of calcium and vitamin D helps maintain peak bone mass. The next stage is bone resorption, associated with higher activity of osteoclasts than osteoblasts, which results in a decreased bone mass and increased risk of fracture [5].

It is vital to notice that low vitamin D concentration causes hyperparathyroidism and decreases intestinal absorption of calcium, leading to bone resorption [10,11]. Vitamin D deficiency is associated with osteoporosis [12]. Moreover, women with fractures presented higher prevalence of vitamin D deficiency [13].

An important element of osteoporosis prevention is physical activity [14]. Physical activity increases BMD [15]. Additionally, regular exercise increases muscle strength, decreasing risk of fall and fracture [16].

Table 1 and Table 2 show calcium content in selected products and the Recommended Daily Intake of calcium for various age groups.

## 2. Milk and BMD

### 2.1. Breast Milk

Compounds of breast milk may come from three sources: the diet of the mother, stocks storage by mother, and lactocytes [19]. The amount of produced milk was negatively correlated with maternal age and weight gained during pregnancy, but these factors did not affect the content of fat in milk. Diet also did not influence the amount and compounds of milk, especially the content of protein, fat, carbohydrates, iron, and calcium. Additionally, fat-soluble vitamins content depended on a mother’s diet to a smaller extent and water-soluble vitamins to a significant one [20,21].

Lactose is the main carbohydrate of human milk. It is made up of glucose and especially important galactose, which supports the development of the central nervous system. Additionally, breast milk contains oligosaccharides (about 15–23 g/L in colostrum and 1–10 g/L in mature milk) [22].

Supplementation of protein in the mother’s diet did not affect milk composition [23]. The main proteins in breast milk are casein, whey protein and mucin; however, protein content decreases with the child’s age [22].

Fats—mostly triacylglycerols (98%)—are a source of about 44% of total breast milk energy. Additionally, breast milk contains more than 200 various fatty acids (FA). The breast milk of European women contains 35–40% of saturated FA, 45–50% mono-unsaturated FA and 15% poly-unsaturated FA [22]. Moreover, the amount of long-chain FA and free FA is greater in human milk than cow’s milk [21]. Fat in human milk is absorbed at around 92%. It is vital to notice that the amount of cholesterol was lower in breast milk than in cow’s or sheep milk [24]. The composition of FA is dependent on the mother’s diet. Patin et al. have shown that, after consumption of 100g of fish (sardines) three times a week by breastfeeding women, the amount of omega-3 FA in breast milk was raised [25].

The optimum calcium:phosphorus (Ca:P) ratio is between 1:1 and 1:2 [26]. Furthermore, the Ca:P ratio is better in breast milk than cow’s milk (1.4–1.7:1 and 1.24:1, respectively) [27]. Additionally, the Ca:P ratio in cow’s milk is dependent on fat content and is higher in whole milk than skimmed [28].

Breast milk is also a source of immune factors, including Il2 (Interleukin 2), Il4, Il10, IgA (immunoglobulin A) total IgG, or macrophages. There were no significant differences in the amount of immune factors in breast milk from women after exposure to stress. However, two weeks after the stressful situation, the level of cortisol in milk was significantly higher. Moreover, breast milk also contains growth hormones [19,29].

### 2.2. Lactation and BMD

Breastfeeding may affect the BMD of both mother and child. Children who were initially (first six months of life) breastfed and later fed milk formula (up to 2 years old) had higher BMD than children only breastfed or only fed with milk formula (for the first two years of life) [30]. According to Blanco et al., exclusive breastfeeding for the first six months of life was associated with higher BMD in adolescents, when compared with mixed feeding [31]. Additionally, 6-year-old children who were breastfed presented higher BMD than children who were never breastfed. Among breastfed children, the group that was exclusively breastfed for minimum the first four months presented lower BMD and higher bone area (BA) than children who were not breastfed for the first four months [32].

On the other hand, among mothers, a breastfeeding period was negatively correlated with BMD of the lumbar spine. Additionally, the frequency of osteoporosis was higher among women who were breastfeeding for a minimum of 37 months than women who were breastfeeding for a shorter period. However, the age of the mother and number of deliveries did not correlate with BMD [33]. According to Tsvetov et al., a negative correlation between the number of deliveries and BMD was reported [34]. Moreover, breastfeeding for more than 18 months increased vertebral fracture risk more than twice in postmenopausal women [35]. In turn, Cooke-Hubley et al. reported that parity and lactation are not associated with higher risk of decreased BMD, clinical fragility or radiographic vertebral fractures over 10 years [36]. It is vital to notice that absorption of calcium during pregnancy increases, but this does not occur during lactation, and calcium is resorbed from the mother’s bones [37].

### 2.3. Cow’s Milk and Dairy Products and BMD

**Figure 2:**
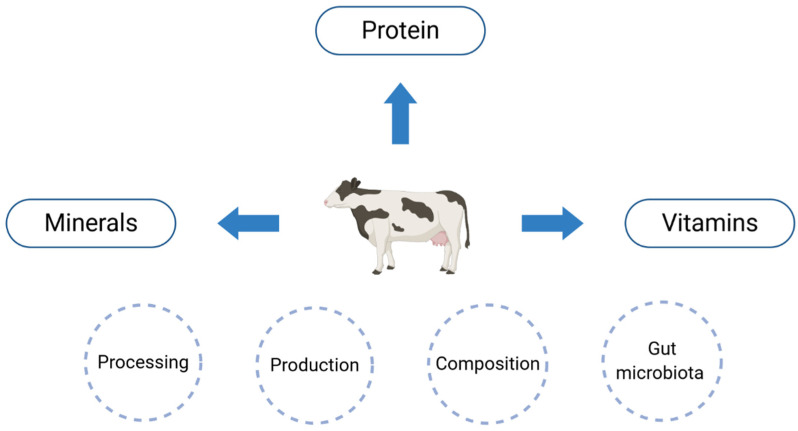
The effect of cow’s milk on bone.

Milk and dairy products contain protein, minerals and vitamins (Figure 2), which may be beneficial for bone health [38]. Cultured dairy products (e.g., yoghurt and kefir) are formed by adding starter cultures, which convert the lactose in milk to lactic acid. For this reason, fermented dairy products may also contain bacteria, which are beneficial for human health [39].

Studies have confirmed that dairy product consumption is essential for human health, especially in the pediatric group. Bone mineral content (BMC) was lower by about 5.6% in women aged 20–49 years who had consumed less than one portion of milk weekly during childhood, when compared with women who had consumed more than one portion. Additionally, low milk consumption during adolescence was associated with a 3% reduction in the BMD and BMC of the hip in adulthood. Among women over 50 years old, there was a non-linear association between milk consumption in childhood and adolescence and BMD and BMC of the hip. Moreover, low milk intake in childhood was linked with two times higher fracture risk [40]. For this reason, osteoporosis is called pediatric disease with geriatric consequences [41]. It is vital to note that children who had avoided milk and had not eaten food fortified with calcium reported fracture before puberty more frequently than children who had consumed cow’s milk [42]. Adults’ height correlated positively with the amount of milk consumed between the ages of 5–12 and 13–17 [43]. Higher consumption of dairy products was associated with higher total BMD among 6-year-old girls and boys. Additionally, positive association occurred between total BMD and intake of a minimum one portion of dairy products daily [44]. Sioen et al. have reported that consumption of dairy products by children (6–12 years old) positively affected total BMC and areal bone mineral density (aBMD) after adjusting for confounding factors [45]. Among young people (18–30 years old), total BMD was lower among people with lower dairy product consumption than subjects with proper intake. There was no significant difference in lumbar spine BMD among groups. It is vital to note that lower intake of dairy products was associated with higher BMI (Body Mass Index) and adipose tissue percentage [46]. On the other hand, as van Dongen et al. have shown, higher intake of milk, milk + yoghurt, and milk + yogurt + cheese was associated with higher trabecular and integral vBMD and VCS among men but not women [47]. Additionally, the positive impact of dairy products on BMD may depend on serum vitamin D levels. Intake of dairy products, fluid dairy and milk was associated with higher BMD of the femoral neck and lumbar spine among subjects with normal 25(OH)D concentration but not in a group with vitamin D deficiency [48]. Among 70-year-old women and men, total dairy product intake was positively associated with trabecular and cortical cross-sectional areas in the tibia and the areal bone mineral density of the radius [49].

On the other hand, as Michaëlsson et al. have reported, dairy product intake was linked with higher mortality in women and men and a higher risk of fracture among women in Sweden [50]. However, it should be mentioned that in Sweden, milk was fortified with vitamin A in the years 1987–1990 and 1997, which may influence the abovementioned results [51]. About 60% of dietary calcium should come from dairy products. Meeting dairy calcium requirements correlated positively with children’s BMD [52]. Meta-analysis has not shown a clear association between the group with an enormous amount of milk intake and risk of osteoporotic fracture and hip fracture. Additionally, results were heterogeneous and did not allow for clear conclusions [53].

### 2.4. Plant Milk (Plant Beverages) and BMD

In the last years, the market availability of plant products, substitutes for cow’s milk, has increased. These products are made from, among others, soybeans, rice, oats, almonds, coconut and are called plant milk or plant beverages. The most similar protein content to cow’s milk occurs in soya beverages. In turn, the content of protein in rice, oats and almond milk is very low. Plant beverages contain a lower amount of saturated fatty acids and do not contain cholesterol. However, producers frequently add fat and sugar to these products, which may increase the risk of metabolic disorders. Moreover, plant beverages contain a lower amount of iodine, potassium, phosphorus and selenium compared with semi-skimmed milk [41].

Data about the differences between the absorption of calcium from dairy and soy products are unclear [54,55]. It is vital to note that the Ca:P ratio in unfortified soya milk is lower than in cow’s milk (2:1 and 1.3:1, respectively). However, calcium fortification changes this ratio for the better (1.8:1) [56]. Nevertheless, calcium and vitamin D fortification of plant beverages is not obligatory in every country [57].

Soy products contain isoflavones, which show an affinity with the estrogen receptor and protects from loss of bone mass. 18-months of intake of cow’s milk fortified with calcium by postmenopausal women increased the BMD of the femoral neck significantly. However, consumption of soy-fortified milk decreased (not significantly) femoral neck BMD [58]. Additionally, intake of cow’s milk with soy isoflavones led to an increase in the level of 25OHD and a decrease in the concentration of bone turnover markers (osteoprotegerin and tartrate-resistant acid phosphatase) [59]. As Lydeking-Olsen et al. have reported among women divided into four groups—consuming soy products, treated with transdermal progesterone (TDP), combined group (consuming soy products and treated with TDP) and control group—BMD and BMC decreased significantly in combined and control groups. BMD and BMC increased in the soy group only, but differences were not significant [60]. An animal study has shown that isoflavones inhibited bone loss in mature female rats with a decreased level of estradiol [61].

An in vitro study has shown that germinated soy germ extracts increased expression of osteocalcin and alkaline phosphatase [62].

Among individuals who weekly drink 1.3 cups of soy milk fortified with calcium, decreased low T-score risk was decreased by 57% when compared with individuals who did not drink soy milk, even if they consumed dairy products [63]. Children are the group who are particularly vulnerable to nutrients deficiencies. Children that consumed plant beverages presented lower serum concentration of vitamin D than children who drank cow’s milk [57].

Cow’s milk is often replaced with plant milk by vegans. According to Ambroszkiewicz et al., people on a vegan diet consume an insufficient amount of calcium and vitamin D, which may lead to osteoporosis [64].

Consumption of unfortified beverages instead of breast milk, cow’s or modified milk may be especially harmful to children in the first year of life because it could lead to the development of rickets, failure in thrive, kwashiorkor, anaemia, metabolic alkalosis, scurvy and hyperoxaluria [65].

Table 3 and Table 4 present the content of nutrients in dairy products and various milks.

## 3. Intolerance and Allergy

### 3.1. Lactose Intolerance

Lactose may be absorbed after decomposing into glucose and galactose by lactase, which is produced in the small intestine. Lactase deficiency—called lactose intolerance (LI)—may cause cramps in the abdomen and pain, diarrhea, and bloating. LI can lead to lower consumption of dairy products [70,71]. LI is genetically conditioned and associated with single polymorphism nucleotide of the *MCM6* gene in 13910CC and 22018GG [72]. Lactose intolerance is different in various countries and affects about 99% of China’s population, 20% of people in the USA, and below 10% of Scandinavia and Netherland inhabitants [73].

Milk and dairy products are the main sources of calcium in many regions around the world. It is vital to note that lactose stimulates calcium absorption in children but not in adults [5]. LI is probably not a direct factor of osteoporosis development; nevertheless, decreased consumption of dairy products due to lactase deficiency and not replacing them with other calcium-rich products may cause decreased BMD.

Among Turkish emigrants in Germany, many presented LI, but it did not affect calcium intake, bone turnover markers or BMD [74]. Meta-analysis has shown that there were no significant differences between subjects with and without lactose LI. The BMD of the total hip was higher among people with lactose tolerance when compared with subjects with LI [73]. BMD was higher among lactose-tolerant subjects with genotype LCT-13910 TT and LCT-13910 CT than lactose-intolerant people with genotype LCT-13910 CC but differences were not significant [75]. For osteoporosis prevention, patients with LI should consume fermented dairy products, lactose-free milk and non-dairy products that are a good source of calcium [72].

### 3.2. Cow’s Milk Allergy

Cow’s milk allergy is a disorder that occurs less frequently than lactose intolerance and is associated with total elimination of milk and dairy products [76]. Patients with IgE-mediated cow’s milk allergy consumed a significantly lower amount of calcium than a control group. Additionally, IgE-mediated cow’s milk allergy increased risk of lower BMD and osteoporosis [77]. Children with cow’s milk allergy had lower BMD in the lumbar spine and femoral neck and consumed less calcium when compared with children without the allergy. The concentration of vitamin D was not different between groups [78]. Therefore, cow’s milk allergy may be associated with development of rickets, and supplementation with calcium and vitamin D may be necessary [79].

## 4. Milk and Dairy Products and Gut Microbiota-Modulation of BMD

Gut microbiota—through the production of biologically active compounds, such as short-chain fatty acids, indole derivatives, polyamines and secondary bile acids—affects not only intestinal cells but also extra-intestinal cells and modulates the immune response. Immune cells correlate with bone cells. Therefore, gut microbiota may affect bone turnover and BMD.

Breast milk is the primary source of nutrition from birth and an important factor for modulating gut microbiota and the further skeletal system. Human milk oligosaccharides (HMU), including galactooligosaccharides (GOS), are important for proper gut microbiota colonization. *Bifidobacterium* ferments GOS and produces short-chain fatty acids [23,80]. *Bifidobacterium* contains lacto-N-biosidase, which facilitates absorption of GOS [81]. According to Matsuki et al., an increase in the number of *Bifidobacterium* increased the amount of HMO in faces. HMO presented a probiotic effect through selective stimulation of *Bifidobacterium* [82]. The study has shown that the amount of *Roseburia, Bifidobacterium* and *Lactobacillus* correlated positively with BMD and T-score.

Additionally, BMD increased proportionally with an increase in the number of *Bifidobacterium* [83]. Breast milk contains many factors that modulate the immune systems of infants. There are immunoglobin (IgA, IgG), lysozyme, lactoferrin, and cytokines regulating immunity (TGF β-Transforming growth factor beta and IL-10), which cause a selection of bacteria colonizing the gastrointestinal tract. IL-10 and TGF-β from breast milk increase immune system toleration for intestine bacteria and promote Il-10 production in infants [84,85]. Additionally, the number of *Bifidobacterium infantis* correlates with the amount of produced IgA and has an anti-inflammatory effect [86]. Breast milk is not sterile and contains about 600 various bacteria spices and cells of bacteria—mainly *Lactobacillus, Weisella, Streptococcus, Lactococcus, Leuconostoc and Enterococcus,* as well as some spices of *Bifidobacterium* [87,88]. It is vital to note that infant formula has a different effect on gut microbiota composition when compared with breast milk. As Brink et al. have reported, in the first years of life, the number of *Bifidobacterium* among infants fed soy formula was 2.6–5 times lower than in breastfed infants [89].

Animal milk contains exosomes, which affect bone formation. Among mice with glucocorticosteroid-induced osteoporosis, administration of exosomes improved BMD when compared with a placebo group (without exosomes administration). Additionally, the amount of *Lactobacillus* decreased in osteoporosis but increased after the use of exosomes. It appears that exosomes isolated from bovine colostrum may be a potential element of the prevention of osteoporosis through modification of gut microbiota and bone remodeling [90].

Fermented dairy products containing probiotic strain may also affect bone metabolism. The randomized study has shown that *Lactobacillus reuteri* decreased bone loss in Swedish women aged 75–80 years with low BMD [91]. Additionally, symbiotics containing *Lactobacillus*, *Bifidobacterium* and FOS (which are components of dairy products) decreased bone turnover among postmenopausal women in Iran [92]. Moreover, *Lactobacillus reuteri* increased serum level of 25OHD among healthy subjects, which affects calcium absorption and may influence on the rising activity of liver 25-hydroxylase [93]. It is vital to notice that consumption of fermented dairy products had a positive effect on bone health independently of total energy, calcium, or protein intakes. This effect was not observed among milk and cheese consumers [94]. Potentially, the probiotics included in these products influence the bone and should be used in patients with lactose intolerance.

Mice fed fermented peptides from kefir had lower trabecular separation and higher BMD among ovariectomy mice. Additionally, animal bone had higher mechanical strength and fracture toughness. Additionally, differentiation of gut microbiota was higher in a group with kefir supplementation than placebo [95].

Probiotic oligosaccharides occur in breast and plant milk [96]. Oligofructose in oat milk has strong prebiotic properties. Consumption of these beverages increases short-chain fatty acid production (butyrate, acetate, propionate), decreases the moderate pH of the colon, increases the faecal mass, and reduces the amount of nitrogen end products and faecal enzymes, which improves immune system function and increases bone mass [97,98]. FOS and GOS increase the percentage of *Bifidobacterium*, which inhibits osteoporosis development [99,100]. Additionally, bacterial fermentation maintains osmotic water retention in the intestine and increases area absorption [101], affecting calcium and phosphorus absorption. Moreover, there is promotion of calcium-binding protein expression and degradation of molecule-binding minerals (among other oxalates, phytic acid). Among healthy postmenopausal women, fermented milk increased the availability of serum isoflavones, which decrease the risk of bone mass loss [102].

Gut microbiota affects bone metabolism through intestinal serotonin (5HT) production. Duodenal enterochromaffin cells are modulated by gut microbiota and are responsible for the synthesis of 5HT. Additionally, short-chain fatty acids increase the production of 5HT [103]. 5HT decreases osteoblasts proliferation through activation of 5-HT1B receptors in preosteoblasts [104]. Regulation of 5HT by gut microbiota may a therapeutic strategy for improving bone health.

Probiotic bacteria may affect bone metabolism. An animal study showed pasteurized *Akkermansia muciniphila* increased parathyroid hormone concentration and the expression of calcium transporters in the kidney [105]. On the other hand, supplementation of probiotics containing 7 bacteria spices decreased the parathyroid hormone in osteopenic patients [92]. Additionally, *Lactocaseibacillus* supplementation decreased high-sensitivity C-reactive protein [106].

Table 5 shows a composition of microorganisms in the selected products.

## 5. Summary—Recommendation for Milk and Dairy Products in the Prevention and Treatment of Osteoporosis

Intake of milk and dairy products is beneficial for every age group but especially for children and adolescents, when the development of bone mass is dynamic. Milk and dairy products are sources of not only high bioavailability calcium but also of vitamin D and proteins. Patients with a lactose intolerance or cow’s milk allergy should avoid or limit milk and dairy product consumption.

Breast milk is an optimal food for infants [96]. Children should be exclusively breastfed for the first six months of their life [20].

Human milk is also preferred food for children with a cow’s milk allergy [115].

Breastfeeding should be recommended for one-year-old children and higher as an element of the diet, if desired by the mother and child [116]. However, it should be reminded that long breastfeeding may affect the BMD of a mother negatively.

Consumption of infant formula between 7 and 12 months of life results in a Ca:P ratio equal to 1.49:1, which complies with recommendations (1:1–1:2) [26].

Homogenized, pasteurized milk (3.25% fat) may be introduced between 9 and 12 months of life. After 9–12 months, sheep’s milk fortified with vitamin D may be introduced as an alternative to cow’s milk [117].

One-year-old children should consume 500 mL (two cups) of milk daily [117].

Skimmed milk (1–2% of fat) may be introduced after the second year of life [117]. According to the World Health Organization, semi-skimmed milk may be introduced after 12 months of life. Skimmed milk is not recommended for children aged less than 12 months because it does not contain essential fatty acids, fat-soluble vitamins, and high potential renal solute load in relation to energy [28].

Plant milk (soy, rice, almond and others) should not be introduced as an alternative to cow’s milk for children under two years of age [117].

Young child formulae are not necessary for children aged 1–3 years, but their implementation is one of the strategies used in order to increase intake of vitamin D, iron and omega-3 fatty acids, which are present in smaller quantities in cow’s milk [116].

Dairy products are a good source of calcium and other nutrients with high bioavailability. Three portions of dairy products may cover the daily need for calcium [51]. According to the recommendation for the population of America, adults should consume three cup-equivalents of fat-free or low-fat (1%) dairy (including milk, yoghurt, cheese or fortified soy beverages) per day [118].

The elderly should not avoid milk and dairy products, because they are a source of high-availability protein, vitamin D, calcium, and phosphorus, which are important for preventing disorders occurring in the elderly, e.g., osteoporosis [119]. It is vital to note that lactose intolerance is often common among older people, and for this group of patients, fermented dairy products (kefir, yoghurt) and lactose-free milk is the best choice.

It is vital to note that cow’s milk and plant beverages are various products, and plant milk cannot be considered as a fully valuable alternative to cow’s milk [41].

According to The National Osteoporosis Foundation, data about the impact of dairy products on bone are moderate [8].

Beneficial modification of gut microbiota due to the consumption of dairy products may increase calcium absorption and the production of short-chain fatty acids and serotonin, which affect bone metabolism directly.

## Figures and Tables

**Table 1:** Calcium content in selected products [17].

Products.	Portion	Calcium Content (mg)
Whole milk	200 mL	236
Semi-skimmed milk	200 mL	240
Skimmed milk	200 mL	244
Sheep milk	200 mL	380
Soy dring (non-enriched)	200 mL	26
Soy drink (calcium-enriched)	200 mL	240
Rice drink	200 mL	22
Almond milk	200 mL	90
Flavoured yoghurt	150 g	197
Natural yoghurt	150 g	207
Hard cheese (e.g., Parmesan, Cheddar)	30 g	240
Fresh cheese (e.g., Ricotta, cottage cheese)	200	138
Mozzarella	60	242

**Table 2:** Recommended Daily Intake of calcium for various age groups [18].

Age	RDI (mg)
0–6 months	200
7–12 months	260
1–3 years	700
4–8 years	1000
9–13 years	1300
14–18 years	1300
19–50 years	1000
51–70 years	
Women	1200
Men	1000
71 years and older	1200
Pregnant and breastfeeding	
Teenagers	1300
Adults	1000

RDI-Recommended Daily Intake.

**Table 3:** Content of nutrients in 100 g of human, cow and plant milk.

	Human [66]	Cow’s [67]	Plant [68]
Fat (g)	3.8	3.7–3.9	0.66–49.2
Proteins (g)	1.0	3.2–3.5	0.59–19.00
Casein (g)	0.3	2.8	0
Carbohydrates (g)	7.0	7.0	27.3–50.0
Lactose (g)	7.0	0.9–4.9	0
Calcium (mg)	34	118	4.0–180.0
Phosphorus (mg)	15	89.6	49.0–1000.0
Sodium (mg)	15	44.5	2.2–140.01
Potassium (mg)	58	150	65.00–2000.0

**Table 4:** Content of macronutrients, calcium, phosphorus and vitamin D in milk and dairy products [69].

	Protein (g)	Fat (g)	Carbohydrates (g)	Calcium (mg)	Phosphorus (mg)	Ca:P Ratio	Wit.D (μg)
Milk, 3.5% fat	3.3	3.5	4.8	118	85	1.38	0.03
Milk, 2% fat	3.4	2.0	4.9	120	86	1.40	0.02
Cream, 18%	2.5	18.0	3.6	99	71	1.39	0.14
Natural yoghurt, 2%	4.3	2.0	6.2	170	122	1.39	0.03
Berries yoghurt	3.7	1.5	8.8	134	96	1.40	0.02
Cheddar cheese	27.1	31.7	0.1	703	487	1.44	0.26
Cottage cheese	12.3	4.3	3.3	80	140	0.57	0.09

**Table 5:** Composition of microorganisms in the selected products.

Milk	Bacteria Strain	References
Breast milk	*Lactocaseibacillus casei* *Limosilactobacillus fermentum* *Lactobacillus gasseri* *Lactobacillus gastricus* *Lactiplantibacillus plantarum* *Lactobacillus reuteri* *Limosilactobacillus rhamnosus* *Ligilactobacillus salivarius* *Lactobacillus vaginalis* *Bifidobacterium breve* *Bifidobacterium longum* *Streptococcus mitis* *Streptococcus salivarius* *Streptococcus parasanguinis* *Enterococcus faecalis* *Enterococcus gallinarum* *Staphylococcus epidermidis* *Staphylococcus lugdunensis* *Staphylococcus aureus* *Staphylococcus haemolyticus* *Staphylococcus pasteuri* *Veillonella atypical* *Lactococcus* *Weissella* *Serratia* *Pseudomonas* *Veillonella* *Leptotrichia* *Prevotella*	[88,107,108]
Cow’s milk	*Lactococcus lactis*, *Streptococcus salivarius ssp. thermophilus,* *Lactobacillus acidophilus,* *Lacticaseibacillus casei* *Limosilactobacillus fermentum.*	[109]
Goast milk	*Lactococcus lactis,* *Lacticaseibacillus paracasei,* *Pediococcus pentosaceus,* *Leuconostoc mesenteroides,* *Streptococcus salivarius subsp. Thermophilus* *Enterococcus faecium*	[110]
Soy milk-added probiotics	*Lactococcus acidophilus* *Lactococcus acidophilus* *Lactococcus casei* *Bifidobacterium longum*	[111,112]
Oats milk-added probiotics	*Lactococcus plantarum 1010*	[113]
Kefir (from cow’s milk)	*Acetobacter orientalis* *Lactococcus lactis* *Lactobacillus gallinarum* *Kazachstania unispora* *Galactomyces candidum* *Geotrichum bryndzae* *Saccharomyces cerevisiae* *Pichia kudriavzevii*	[114]
Kefir (from soy milk)	*Lactococcus lactis* *Kazachstania unispora* *Saccharomyces cerevisiae* *Lactobacillus nagelii* *Lactiplantibacillus plantarum*	[114]

## Data Availability

Not applicable.

## References

[1] Janiszewska M., Kulik T., Dziedzic M., Żołnierczuk-Kieliszek D., Barańska A. (2015). Osteoporosis as a Social Problem- Pathogenesis, Symptoms and Risk Factors of Postmenopausal Osteoporosis. Probl. Hig. Epidemiol..

[2] Pouresmaeili F., Kamalidehghan B., Kamarehei M., Goh Y.M. (2018). A Comprehensive Overview on Osteoporosis and Its Risk Factors. Ther. Clin. Risk Manag..

[3] Ratajczak A.E., Rychter A.M., Zawada A., Dobrowolska A., Krela-Kaźmierczak I. (2020). Nutrients in the Prevention of Osteoporosis in Patients with Inflammatory Bowel Diseases. Nutrients.

[4] Rosen C.J. (2020). The Epidemiology and Pathogenesis of Osteoporosis.

[5] Hodges J.K., Cao S., Cladis D.P., Weaver C.M. (2019). Lactose Intolerance and Bone Health: The Challenge of Ensuring Adequate Calcium Intake. Nutrients.

[6] Gordon C.M., Zemel B.S., Wren T.A.L., Leonard M.B., Bachrach L.K., Rauch F., Gilsanz V., Rosen C.J., Winer K.K. (2017). The Determinants of Peak Bone Mass. J. Pediatrics.

[7] Osteoporosis: Peak Bone Mass in Women|NIH Osteoporosis and Related Bone Diseases National Resource Center. https://www.bones.nih.gov/health-info/bone/osteoporosis/bone-mass.

[8] Weaver C.M., Gordon C.M., Janz K.F., Kalkwarf H.J., Lappe J.M., Lewis R., O’Karma M., Wallace T.C., Zemel B.S. (2016). The National Osteoporosis Foundation’s Position Statement on Peak Bone Mass Development and Lifestyle Factors: A Systematic Review and Implementation Recommendations. Osteoporos. Int..

[9] McGuigan F.E.A., Murray L., Gallagher A., Davey-Smith G., Neville C.E., Van’t Hof R., Boreham C., Ralston S.H. (2002). Genetic and Environmental Determinants of Peak Bone Mass in Young Men and Women. J. Bone Miner. Res..

[10] Muscogiuri G., Barrea L., Altieri B., Di Somma C., Bhattoa H.P., Laudisio D., Duval G.T., Pugliese G., Annweiler C., Orio F. (2019). Calcium and Vitamin D Supplementation. Myths and Realities with Regard to Cardiovascular Risk. Curr. Vasc. Pharmacol..

[11] Fischer V., Haffner-Luntzer M., Amling M., Ignatius A. (2018). Calcium and Vitamin D in Bone Fracture Healing and Post-Traumatic Bone Turnover. Eur. Cell Mater..

[12] Dadra A., Aggarwal S., Kumar P., Kumar V., Dibar D.P., Bhadada S.K. (2019). High Prevalence of Vitamin D Deficiency and Osteoporosis in Patients with Fragility Fractures of Hip: A Pilot Study. J. Clin. Orthop. Trauma.

[13] Lee J.S., Kim J.W. (2018). Prevalence of Vitamin D Deficiency in Postmenopausal High- and Low-Energy Fracture Patient. Arch. Osteoporos..

[14] Warburton D.E.R., Nicol C.W., Bredin S.S.D. (2006). Health Benefits of Physical Activity: The Evidence. CMAJ.

[15] Maggio A.B.R., Rizzoli R.R., Marchand L.M., Ferrari S., Beghetti M., Farpour-Lambert N.J. (2012). Physical Activity Increases Bone Mineral Density in Children with Type 1 Diabetes. Med. Sci. Sports Exerc..

[16] Chan D.-C., Chang C.-B., Han D.-S., Hong C.-H., Hwang J.-S., Tsai K.-S., Yang R.-S. (2018). Effects of Exercise Improves Muscle Strength and Fat Mass in Patients with High Fracture Risk: A Randomized Control Trial. J. Formos. Med. Assoc..

[17] Calcium Content of Common Foods | International Osteoporosis Foundation. https://www.osteoporosis.foundation/patients/prevention/calcium-content-of-common-foods.

[18] Office of Dietary Supplements-Calcium. https://ods.od.nih.gov/factsheets/Calcium-Consumer/.

[19] Ballard O., Morrow A.L. (2013). Human Milk Composition: Nutrients and Bioactive Factors. Pediatr. Clin. North. Am..

[20] Hytten F.E. (1954). Clinical and Chemical Studies in Human Lactation. VIII. Relationship of the Age, Physique, and Nutritional Status of the Mother to the Yield and Composition of Her Milk. Br. Med. J..

[21] Thomson A.M., Black A.E. (1975). Nutritional Aspects of Human Lactation. Bull. World Health Organ..

[22] Mosca F., Giannì M.L. (2017). Human Milk: Composition and Health Benefits. Pediatr. Med. Chir..

[23] Gopalan C. (1962). Effect of Nutrition on Pregnancy and Lactation. Bull. World Health Organ..

[24] Pietrzak-Fiećko R., Kamelska-Sadowska A.M. (2020). The Comparison of Nutritional Value of Human Milk with Other Mammals’ Milk. Nutrients.

[25] Patin R.V., Vítolo M.R., Valverde M.A., Carvalho P.O., Pastore G.M., Lopez F.A. (2006). The Influence of Sardine Consumption on the Omega-3 Fatty Acid Content of Mature Human Milk. J. Pediatr..

[26] Loughrill E., Wray D., Christides T., Zand N. (2017). Calcium to Phosphorus Ratio, Essential Elements and Vitamin D Content of Infant Foods in the UK: Possible Implications for Bone Health. Matern. Child. Nutr..

[27] Mahdi A.A., Brown R.B., Razzaque M.S. (2015). Osteoporosis in Populations with High Calcium Intake: Does Phosphate Toxicity Explain the Paradox?. Ind. J. Clin. Biochem..

[28] Burgess K., Muehlhoff E., Bennett A., McMahon D. (2013). Milk and Dairy Products in Human Nutrition.

[29] Aparicio M., Browne P.D., Hechler C., Beijers R., Rodríguez J.M., de Weerth C., Fernández L. (2020). Human Milk Cortisol and Immune Factors over the First Three Postnatal Months: Relations to Maternal Psychosocial Distress. PLoS ONE.

[30] Al-Agha A.E., Kabli Y.O., AlBeiruty M.G., Milyani A.A. (2019). Determinants of Bone Mineral Density through Quantitative Ultrasound Screening of Healthy Children Visiting Ambulatory Paediatric Clinics. Saudi Med. J..

[31] Blanco E., Burrows R., Reyes M., Lozoff B., Gahagan S., Albala C. (2017). Breastfeeding as the Sole Source of Milk for 6 Months and Adolescent Bone Mineral Density. Osteoporos. Int..

[32] van den Hooven E.H., Gharsalli M., Heppe D.H.M., Raat H., Hofman A., Franco O.H., Rivadeneira F., Jaddoe V.W.V. (2016). Associations of Breast-Feeding Patterns and Introduction of Solid Foods with Childhood Bone Mass: The Generation R Study. Br. J. Nutr..

[33] Hwang I.R., Choi Y.K., Lee W.K., Kim J.G., Lee I.K., Kim S.W., Park K.G. (2016). Association between Prolonged Breastfeeding and Bone Mineral Density and Osteoporosis in Postmenopausal Women: KNHANES 2010–2011. Osteoporos. Int..

[34] Tsvetov G., Levy S., Benbassat C., Shraga-Slutzky I., Hirsch D. (2014). Influence of Number of Deliveries and Total Breast-Feeding Time on Bone Mineral Density in Premenopausal and Young Postmenopausal Women. Maturitas.

[35] Bolzetta F., Veronese N., De Rui M., Berton L., Carraro S., Pizzato S., Girotti G., De Ronch I., Manzato E., Coin A. (2014). Duration of Breastfeeding as a Risk Factor for Vertebral Fractures. Bone.

[36] Cooke-Hubley S., Gao Z., Mugford G., Kaiser S.M., Goltzman D., Leslie W.D., Davison K.S., Brown J.P., Probyn L., Lentle B. (2019). Parity and Lactation Are Not Associated with Incident Fragility Fractures or Radiographic Vertebral Fractures over 16 Years of Follow-up: Canadian Multicentre Osteoporosis Study (CaMos). Arch. Osteoporos..

[37] Kovacs C.S., Feingold K.R., Anawalt B., Boyce A., Chrousos G., de Herder W.W., Dungan K., Grossman A., Hershman J.M., Hofland J., Kaltsas G. (2000). Calcium and Phosphate Metabolism and Related Disorders during Pregnancy and Lactation. Endotext.

[38] Tunick M.H., Van Hekken D.L. (2015). Dairy Products and Health: Recent Insights. J. Agric. Food Chem..

[39] Aryana K.J., Olson D.W. (2017). A 100-Year Review: Yogurt and Other Cultured Dairy Products. J. Dairy Sci..

[40] Kalkwarf H.J., Khoury J.C., Lanphear B.P. (2003). Milk Intake during Childhood and Adolescence, Adult Bone Density, and Osteoporotic Fractures in US Women. Am. J. Clin. Nutr..

[41] Thorning T.K., Raben A., Tholstrup T., Soedamah-Muthu S.S., Givens I., Astrup A. (2016). Milk and Dairy Products: Good or Bad for Human Health? An Assessment of the Totality of Scientific Evidence. Food Nutr. Res..

[42] Goulding A., Rockell J.E.P., Black R.E., Grant A.M., Jones I.E., Williams S.M. (2004). Children Who Avoid Drinking Cow’s Milk Are at Increased Risk for Prepubertal Bone Fractures. J. Am. Diet. Assoc..

[43] Wiley A.S. (2005). Does Milk Make Children Grow? Relationships between Milk Consumption and Height in NHANES 1999–2002. Am. J. Hum. Biol..

[44] Bielemann R.M., dos S Vaz J., Domingues M.R., Matijasevich A., Santos I.S., Ekelund U., Horta B.L. (2018). Are Consumption of Dairy Products and Physical Activity Independently Related to Bone Mineral Density of 6-Year-Old Children? Longitudinal and Cross-Sectional Analyses in a Birth Cohort from Brazil. Public Health Nutr..

[45] Sioen I., Michels N., Polfliet C., De Smet S., D’Haese S., Roggen I., Deschepper J., Goemaere S., Valtueña J., De Henauw S. (2015). The Influence of Dairy Consumption, Sedentary Behaviour and Physical Activity on Bone Mass in Flemish Children: A Cross-Sectional Study. BMC Public Health.

[46] Torres-Costoso A., López-Muñoz P., Ferri-Morales A., Bravo-Morales E., Martínez-Vizcaíno V., Garrido-Miguel M. (2019). Body Mass Index, Lean Mass, and Body Fat Percentage as Mediators of the Relationship between Milk Consumption and Bone Health in Young Adults. Nutrients.

[47] van Dongen L.H., Kiel D.P., Soedamah-Muthu S.S., Bouxsein M.L., Hannan M.T., Sahni S. (2018). Higher Dairy Food Intake Is Associated With Higher Spine Quantitative Computed Tomography (QCT) Bone Measures in the Framingham Study for Men But Not Women. J. Bone Miner. Res..

[48] Mangano K.M., Noel S.E., Sahni S., Tucker K.L. (2019). Higher Dairy Intakes Are Associated with Higher Bone Mineral Density among Adults with Sufficient Vitamin D Status: Results from the Boston Puerto Rican Osteoporosis Study. J. Nutr..

[49] Hallkvist O.M., Johansson J., Nordström A., Nordström P., Hult A. (2018). Dairy Product Intake and Bone Properties in 70-Year-Old Men and Women. Arch. Osteoporos..

[50] Michaëlsson K., Wolk A., Langenskiöld S., Basu S., Warensjö Lemming E., Melhus H., Byberg L. (2014). Milk Intake and Risk of Mortality and Fractures in Women and Men: Cohort Studies. BMJ.

[51] Rozenberg S., Body J.-J., Bruyère O., Bergmann P., Brandi M.L., Cooper C., Devogelaer J.-P., Gielen E., Goemaere S., Kaufman J.-M. (2016). Effects of Dairy Products Consumption on Health: Benefits and Beliefs—Commentary from the Belgian Bone Club and the European Society for Clinical and Economic Aspects of Osteoporosis, Osteoarthritis and Musculoskeletal Diseases. Calcif. Tissue Int..

[52] Infante D., Tormo R. (2000). Risk of Inadequate Bone Mineralization in Diseases Involving Long-Term Suppression of Dairy Products. J. Pediatric Gastroenterol. Nutr..

[53] Matía-Martín P., Torrego-Ellacuría M., Larrad-Sainz A., Fernández-Pérez C., Cuesta-Triana F., Rubio-Herrera M.Á. (2019). Effects of Milk and Dairy Products on the Prevention of Osteoporosis and Osteoporotic Fractures in Europeans and Non-Hispanic Whites from North America: A Systematic Review and Updated Meta-Analysis. Adv. Nutr..

[54] Tang A.L., Walker K.Z., Wilcox G., Strauss B.J., Ashton J.F., Stojanovska L. (2010). Calcium Absorption in Australian Osteopenic Post-Menopausal Women: An Acute Comparative Study of Fortified Soymilk to Cows’ Milk. Asia Pac. J. Clin. Nutr..

[55] Heaney R.P., Dowell M.S., Rafferty K., Bierman J. (2000). Bioavailability of the Calcium in Fortified Soy Imitation Milk, with Some Observations on Method. Am. J. Clin. Nutr..

[56] Geiker N.R.W., Mølgaard C., Iuliano S., Rizzoli R., Manios Y., van Loon L.J.C., Lecerf J.-M., Moschonis G., Reginster J.-Y., Givens I. (2020). Impact of Whole Dairy Matrix on Musculoskeletal Health and Aging–Current Knowledge and Research Gaps. Osteoporos. Int..

[57] Lee G.J., Birken C.S., Parkin P.C., Lebovic G., Chen Y., L’Abbé M.R., Maguire J.L. (2014). TARGet Kids! Collaboration Consumption of Non-Cow’s Milk Beverages and Serum Vitamin D Levels in Early Childhood. CMAJ.

[58] Gui J.-C., Brašić J.R., Liu X.-D., Gong G.-Y., Zhang G.-M., Liu C.-J., Gao G.-Q. (2012). Bone Mineral Density in Postmenopausal Chinese Women Treated with Calcium Fortification in Soymilk and Cow’s Milk. Osteoporos. Int..

[59] García-Martín A., Quesada Charneco M., Alvárez Guisado A., Jiménez Moleón J.J., Fonollá Joya J., Muñoz-Torres M. (2012). Effect of milk product with soy isoflavones on quality of life and bone metabolism in postmenopausal Spanish women: Randomized trial. Med. Clin..

[60] Lydeking-Olsen E., Beck-Jensen J.-E., Setchell K.D.R., Holm-Jensen T. (2004). Soymilk or Progesterone for Prevention of Bone Loss—A 2 Year Randomized, Placebo-Controlled Trial. Eur. J. Nutr..

[61] Yanaka K., Higuchi M., Ishimi Y. (2019). Anti-Osteoporotic Effect of Soy Isoflavones Intake on Low Bone Mineral Density Caused by Voluntary Exercise and Food Restriction in Mature Female Rats. J. Nutr. Sci. Vitam..

[62] Choi C.-W., Choi S.-W., Kim H.-J., Lee K.-S., Kim S.-H., Kim S.-L., Do S.H., Seo W.-D. (2018). Germinated Soy Germ with Increased Soyasaponin Ab Improves BMP-2-Induced Bone Formation and Protects against in Vivo Bone Loss in Osteoporosis. Sci. Rep..

[63] Matthews V.L., Knutsen S.F., Beeson W.L., Fraser G.E. (2011). Soy Milk and Dairy Consumption Are Independently Associated with Ultrasound Attenuation of the Heel Bone among Postmenopausal Women: The Adventist Health Study-2 (AHS-2). Nutr. Res..

[64] Ambroszkiewicz J., Klemarczyk W., Gajewska J., Chełchowska M., Franek E., Laskowska-Klita T. (2010). The Influence of Vegan Diet on Bone Mineral Density and Biochemical Bone Turnover Markers. Pediatr. Endocrinol. Diabetes Metab..

[65] Vitoria I. (2017). The Nutritional Limitations of Plant-Based Beverages in Infancy and Childhood. Nutr. Hosp..

[66] Kowalska D., Gruczyńska E., Bryś J. (2015). Mother’s Milk—First Food in Human Life. Probl. Hig. Epidemiol..

[67] Guetouache M., Guessas B., Medjekal S. (2014). Composition and Nutritional Value of Raw Milk. Issues Biol. Sci. Pharm. Res..

[68] Paul A.A., Kumar S., Kumar V., Sharma R. (2020). Milk Analog: Plant Based Alternatives to Conventional Milk, Production, Potential and Health Concerns. Crit. Rev. Food Sci. Nutr..

[69] Kunachowicz H., Przygoda B., Nadolna I., Iwanow K. (2017). Tabele Skłądu I Wartości Odżywczej Żywności.

[70] Heaney R.P. (2013). Dairy Intake, Dietary Adequacy, and Lactose Intolerance12. Adv. Nutr..

[71] Keith J.N., Nicholls J., Reed A., Kafer K., Miller G.D. (2011). The Prevalence of Self-Reported Lactose Intolerance and the Consumption of Dairy Foods among African American Adults Are Less than Expected. J. Natl. Med. Assoc..

[72] Ratajczak A.E., Rychter A.M., Zawada A., Dobrowolska A., Krela-Kaźmierczak I. (2020). Lactose Intolerance in Patients with Inflammatory Bowel Diseases and Dietary Management in Prevention of Osteoporosis. Nutrition.

[73] Treister-Goltzman Y., Friger M., Peleg R. (2018). Does Primary Lactase Deficiency Reduce Bone Mineral Density in Postmenopausal Women? A Systematic Review and Meta-Analysis. Osteoporos. Int..

[74] Klemm P., Dischereit G., Lange U. (2019). Adult Lactose Intolerance, Calcium Intake, Bone Metabolism and Bone Density in German-Turkish Immigrants. J. Bone Miner. Metab..

[75] Mnich B., Spinek A.E., Chyleński M., Sommerfeld A., Dabert M., Juras A., Szostek K. (2018). Analysis of LCT-13910 Genotypes and Bone Mineral Density in Ancient Skeletal Materials. PLoS ONE.

[76] Domínguez-García V., Flores-Merino M.V., Morales-Romero J., Bedolla-Pulido A., Mariscal-Castro J., Bedolla-Barajas M. (2019). Allergy to cow’s milk protein, or lactose intolerance: A cross-sectional study in university students. Rev. Alerg. Mex..

[77] Nachshon L., Goldberg M.R., Schwartz N., Sinai T., Amitzur-Levy R., Elizur A., Eisenberg E., Katz Y. (2014). Decreased Bone Mineral Density in Young Adult IgE-Mediated Cow’s Milk-Allergic Patients. J. Allergy Clin. Immunol..

[78] Mailhot G., Perrone V., Alos N., Dubois J., Delvin E., Paradis L., Des Roches A. (2016). Cow’s Milk Allergy and Bone Mineral Density in Prepubertal Children. Pediatrics.

[79] Yu J.W., Pekeles G., Legault L., McCusker C.T. (2006). Milk Allergy and Vitamin D Deficiency Rickets: A Common Disorder Associated with an Uncommon Disease. Ann. Allergy Asthma Immunol..

[80] Marcobal A., Barboza M., Froehlich J.W., Block D.E., German J.B., Lebrilla C.B., Mills D.A. (2010). Consumption of Human Milk Oligosaccharides by Gut-Related Microbes. J. Agric. Food Chem..

[81] Sakurama H., Kiyohara M., Wada J., Honda Y., Yamaguchi M., Fukiya S., Yokota A., Ashida H., Kumagai H., Kitaoka M. (2013). Lacto- *N* -Biosidase Encoded by a Novel Gene of *Bifidobacterium Longum* Subspecies *Longum* Shows Unique Substrate Specificity and Requires a Designated Chaperone for Its Active Expression. J. Biol. Chem..

[82] Matsuki T., Yahagi K., Mori H., Matsumoto H., Hara T., Tajima S., Ogawa E., Kodama H., Yamamoto K., Yamada T. (2016). A Key Genetic Factor for Fucosyllactose Utilization Affects Infant Gut Microbiota Development. Nat. Commun..

[83] Li C., Huang Q., Yang R., Dai Y., Zeng Y., Tao L., Li X., Zeng J., Wang Q. (2019). Gut Microbiota Composition and Bone Mineral Loss—Epidemiologic Evidence from Individuals in Wuhan, China. Osteoporos. Int..

[84] Levast B., Li Z., Madrenas J. (2015). The Role of IL-10 in Microbiome-Associated Immune Modulation and Disease Tolerance. Cytokine.

[85] Brandtzaeg P. (2003). Mucosal Immunity: Integration between Mother and the Breast-Fed Infant. Vaccine.

[86] Chichlowski M., De Lartigue G., German J.B., Raybould H.E., Mills D.A. (2012). Bifidobacteria Isolated From Infants and Cultured on Human Milk Oligosaccharides Affect Intestinal Epithelial Function. J. Pediatric Gastroenterol. Nutr..

[87] Jeurink P.V., van Bergenhenegouwen J., Jiménez E., Knippels L.M.J., Fernández L., Garssen J., Knol J., Rodríguez J.M., Martín R. (2013). Human Milk: A Source of More Life than We Imagine. Benef. Microbes.

[88] Jost T., Lacroix C., Braegger C., Chassard C. (2013). Assessment of Bacterial Diversity in Breast Milk Using Culture-Dependent and Culture-Independent Approaches. Br. J. Nutr..

[89] Brink L.R., Mercer K.E., Piccolo B.D., Chintapalli S.V., Elolimy A., Bowlin A.K., Matazel K.S., Pack L., Adams S.H., Shankar K. (2020). Neonatal Diet Alters Fecal Microbiota and Metabolome Profiles at Different Ages in Infants Fed Breast Milk or Formula. Am. J. Clin. Nutr..

[90] Yun B., Maburutse B.E., Kang M., Park M.R., Park D.J., Kim Y., Oh S. (2020). Short Communication: Dietary Bovine Milk–Derived Exosomes Improve Bone Health in an Osteoporosis-Induced Mouse Model. J. Dairy Sci..

[91] Nilsson A.G., Sundh D., Bäckhed F., Lorentzon M. (2018). *Lactobacillus Reuteri* Reduces Bone Loss in Older Women with Low Bone Mineral Density: A Randomized, Placebo-Controlled, Double-Blind, Clinical Trial. J. Intern. Med..

[92] Jafarnejad S., Djafarian K., Fazeli M.R., Yekaninejad M.S., Rostamian A., Keshavarz S.A. (2017). Effects of a Multispecies Probiotic Supplement on Bone Health in Osteopenic Postmenopausal Women: A Randomized, Double-Blind, Controlled Trial. Null.

[93] Jones M.L., Martoni C.J., Prakash S. (2013). Oral Supplementation With Probiotic *L. Reuteri* NCIMB 30242 Increases Mean Circulating 25-Hydroxyvitamin D: A Post Hoc Analysis of a Randomized Controlled Trial. J. Clin. Endocrinol. Metab..

[94] Biver E., Durosier-Izart C., Merminod F., Chevalley T., van Rietbergen B., Ferrari S.L., Rizzoli R. (2018). Fermented Dairy Products Consumption Is Associated with Attenuated Cortical Bone Loss Independently of Total Calcium, Protein, and Energy Intakes in Healthy Postmenopausal Women. Osteoporos. Int..

[95] Tu M.-Y., Han K.-Y., Chang G.R.-L., Lai G.-D., Chang K.-Y., Chen C.-F., Lai J.-C., Lai C.-Y., Chen H.-L., Chen C.-M. (2020). Kefir Peptides Prevent Estrogen Deficiency-Induced Bone Loss and Modulate the Structure of the Gut Microbiota in Ovariectomized Mice. Nutrients.

[96] Whisner C.M., Castillo L.F. (2018). Prebiotics, Bone and Mineral Metabolism. Calcif. Tissue Int..

[97] Markowiak P., Śliżewska K. (2017). Effects of Probiotics, Prebiotics, and Synbiotics on Human Health. Nutrients.

[98] Lee Y.K., Salminen S. (2008). Handbook of Probiotics and Prebiotics.

[99] Bornet F.R.J., Brouns F., Tashiro Y., Duvillier V. (2002). Nutritional Aspects of Short-Chain Fructooligosaccharides: Natural Occurrence, Chemistry, Physiology and Health Implications. Dig. Liver Dis..

[100] Vulevic J., Juric A., Walton G.E., Claus S.P., Tzortzis G., Toward R.E., Gibson G.R. (2015). Influence of Galacto-Oligosaccharide Mixture (B-GOS) on Gut Microbiota, Immune Parameters and Metabonomics in Elderly Persons. Br. J. Nutr..

[101] Scholz-Ahrens K.E., Ade P., Marten B., Weber P., Timm W., Aςil Y., Glüer C.-C., Schrezenmeir J. (2007). Prebiotics, Probiotics, and Synbiotics Affect Mineral Absorption, Bone Mineral Content, and Bone Structure. J. Nutr..

[102] Timan P., Rojanasthien N., Manorot M., Sangdee C., Teekachunhatean S. (2014). Effect of Synbiotic Fermented Milk on Oral Bioavailability of Isoflavones in Postmenopausal Women. Null.

[103] Yano J.M., Yu K., Donaldson G.P., Shastri G.G., Ann P., Ma L., Nagler C.R., Ismagilov R.F., Mazmanian S.K., Hsiao E.Y. (2015). Indigenous Bacteria from the Gut Microbiota Regulate Host Serotonin Biosynthesis. Cell.

[104] Kode A., Mosialou I., Silva B.C., Rached M.-T., Zhou B., Wang J., Townes T.M., Hen R., DePinho R.A., Guo X.E. (2012). FOXO1 Orchestrates the Bone-Suppressing Function of Gut-Derived Serotonin. J. Clin. Investig..

[105] Lawenius L., Scheffler J.M., Gustafsson K.L., Henning P., Nilsson K.H., Colldén H., Islander U., Plovier H., Cani P.D., de Vos W.M. (2020). Pasteurized Akkermansia Muciniphila Protects from Fat Mass Gain but Not from Bone Loss. Am. J. Physiol. Endocrinol. Metab..

[106] Lei M., Guo C., Wang D., Zhang C., Hua L. (2017). The Effect of Probiotic Lactobacillus Casei Shirota on Knee Osteoarthritis: A Randomised Double-Blind, Placebo-Controlled Clinical Trial. Benef. Microbes.

[107] Soto A., Martín V., Jiménez E., Mader I., Rodríguez J.M., Fernández L. (2014). Lactobacilli and Bifidobacteria in Human Breast Milk: Influence of Antibiotherapy and Other Host and Clinical Factors. J. Pediatr. Gastroenterol. Nutr..

[108] Cabrera-Rubio R., Collado M.C., Laitinen K., Salminen S., Isolauri E., Mira A. (2012). The Human Milk Microbiome Changes over Lactation and Is Shaped by Maternal Weight and Mode of Delivery. Am. J. Clin. Nutr..

[109] Igras S. (2012). Characteristics of milk of various animal and human species. J. NutriLife.

[110] Pisano M.B., Deplano M., Fadda M.E., Cosentino S. (2019). Microbiota of Sardinian Goat’s Milk and Preliminary Characterization of Prevalent LAB Species for Starter or Adjunct Cultures Development. BioMed Res. Int..

[111] Yeo S.-K., Liong M.-T. (2010). Angiotensin I-Converting Enzyme Inhibitory Activity and Bioconversion of Isoflavones by Probiotics in Soymilk Supplemented with Prebiotics. Null.

[112] Zielińska D. (2005). Selecting Suitable Bacterial Strains of Lactobacillus and Identifying Soya Drink Fermentation Conditions. Żywność. Nauka. Technologia. Jakość.

[113] Gupta S., Cox S., Abu-Ghannam N. (2010). Process Optimization for the Development of a Functional Beverage Based on Lactic Acid Fermentation of Oats. Biochem. Eng. J..

[114] Gamba R.R., Yamamoto S., Abdel-Hamid M., Sasaki T., Michihata T., Koyanagi T., Enomoto T. (2020). Chemical, Microbiological, and Functional Characterization of Kefir Produced from Cow’s Milk and Soy Milk. Int. J. Microbiol..

[115] Vandenplas Y., Brueton M., Dupont C., Hill D., Isolauri E., Koletzko S., Oranje A.P., Staiano A. (2007). Guidelines for the Diagnosis and Management of Cow’s Milk Protein Allergy in Infants. Arch. Dis. Child..

[116] Hojsak I., Bronsky J., Campoy C., Domellöf M., Embleton N., Fidler Mis N., Hulst J., Indrio F., Lapillonne A., Mølgaard C. (2018). Young Child Formula: A Position Paper by the ESPGHAN Committee on Nutrition. J. Pediatric Gastroenterol. Nutr..

[117] Services A.H. Healthy Infants and Young Children. https://www.albertahealthservices.ca/info/Page8567.aspx.

[118] 2015–2020 Dietary Guidelines|Health.Gov. https://health.gov/our-work/food-nutrition/2015-2020-dietary-guidelines/guidelines/#subnav-3.

[119] Marangoni F., Pellegrino L., Verduci E., Ghiselli A., Bernabei R., Calvani R., Cetin I., Giampietro M., Perticone F., Piretta L. (2019). Cow’s Milk Consumption and Health: A Health Professional’s Guide. J. Am. Coll. Nutr..

